# Genomic characterization of extended-spectrum β-lactamase-producing *Enterobacterales* isolated from abdominal surgical patients

**DOI:** 10.1017/S0950268824000578

**Published:** 2024-04-12

**Authors:** Sumalee Kondo, Worawich Phornsiricharoenphant, Lalita Na-rachasima, Pimonwan Phokhaphan, Wuthiwat Ruangchai, Prasit Palittapongarnpim, Anucha Apisarnthanarak

**Affiliations:** 1 Faculty of Medicine, Thammasat University, Pathum Thani, Thailand; 2 National Center for Genetic Engineering and Biotechnology, National Science and Technology Development Agency, Pathum Thani, Thailand; 3 Graduate School, Faculty of Medicine, Thammasat University, Pathum Thani, Thailand; 4 Pornchai Matangkasombut Center for Microbial Genomics, Mahidol University, Bangkok, Thailand

**Keywords:** abdominal surgical patients, *bla* genes, extended-spectrum β-lactamase-producing *Enterobacterales*, faecal ESBL carriage, resistance genes

## Abstract

Rectal swabs of 104 patients who underwent abdominal surgery were screened for ESBL producers. Sequence types (STs) and resistance genes were identified by whole-genome sequencing of 46 isolates from 17 patients. All but seven isolates were assigned to recognized STs. While 18 ESBL-producing *E. coli* (EPEC) strains were of unique STs, ESBL-producing *K. pneumoniae* (EPKP) strains were mainly ST14 or ST15. Eight patients harboured strains of the same ST before and after abdominal surgery. The most prevalent resistant genes in *E. coli* were *bla*_EC_ (69.57%), *bla*_CTX-M_ (65.22%), and *bla*_TEM_ (36.95%), while *bla*_SHV_ was present in only *K. pneumoniae* (41.30%). Overall, genes encoding β-lactamases of classes A (*bla*_CTX-M_, *bla*_TEM_, *bla*_Z_), C (*bla*_SHV_, *bla*_MIR_, and *bla*_DHA_), and D (*bla*_OXA_) were identified, the most prevalent variants being *bla*_CTX-M-15_, *bla*_TEM-1B_, *bla*_SHV-28_, and *bla*_OXA-1._ Interestingly, *bla*_CMY-2_, the most common pAmpC β-lactamase genes reported worldwide, and mobile colistin resistance genes, *mcr-10-1*, were also identified. The presence of *bla*_CMY-2_ and *mcr-10-1* is concerning as they may constitute a potentially high risk of pan-resistant post-surgical infections. It is imperative that healthcare professionals monitor intra-abdominal surgical site infections rigorously to prevent transmission of faecal ESBL carriage in high-risk patients.

## Introduction

Extended-spectrum β-lactamase-producing *Enterobacterales* (ESBL-PE) are a serious global health concern for transmission of multidrug-resistant organisms, particularly *Escherichia coli* and *Klebsiella pneumoniae.* Hospital-acquired infections, including surgical site infections caused by ESBL-PE, are associated with considerable morbidity and mortality [1]. Contaminated surgical wounds and medical devices, along with admission to hospital more than 24 hours before surgery, were identified as the most statistically significant risk factors in a recent study [2] and underline the need for preventive measures to improve surgical outcomes [3].

We investigated whether faecal carriage of ESBL organisms in patients before abdominal surgery constituted a source of post-surgical infections in these subjects. Isolates recovered from rectal swabs of 104 patients 1 day before and up to 3 days post-surgery were characterized by molecular characteristics and ESBL resistance genes to confirm prior colonization with and persistence of ESBL-PE strains.

## Materials and methods

Rectal swabs were cultured on selective CHROMagar ESBL (SIGMA-ALDRICH, St. Louis, USA) and MacConkey agar (Becton, Dickinson, Sparks, USA). Isolates were identified to the species level by standard biochemical tests. Combination disk diffusion tests [3] were performed for phenotypic confirmation of the presence of ESBLs using appropriate control reference strains. ESBL production was confirmed by an increase of ≥5 mm using combination disks of cetazidime (30 μg)/clavulanate (10 μg) or cefotaxime (30 μg)/clavulanate (10 μg) compared against CAZ (30 μg), or CTX (30µg) alone. *K. pneumoniae* ATCC 700603 and *E. coli* ATCC 25922 were included as ESBL-positive and ESBL-negative controls.

## Detection of resistance genes by whole-genome sequencing

Of the 104 patients who underwent abdominal surgery from July 2018 to March 2019, 31 were positive for ESBL-producing *E. coli* and *K. pneumoniae* in their faecal flora. From the 17 patients who yielded ESBL-PE organisms on pre- and post-surgical screening, 46 isolates were recovered, except for one patient (number 30) where ESBL-KP and KP^R^ phenotypes were found only in the post-operation specimen. The 46 selected isolates were subjected to whole-genome sequence analysis.

The quantity and quality of DNA extracts were determined by gel electrophoresis and fluorescent measurement by Qubit assay (Thermo Fisher Scientific, Vilnius, Lithuania). DNA libraries were constructed using MGIEasy FS DNA library kit and sequenced with a DNBSEQ-G400 sequencer (MGI Tech, Shenzhen, China). All isolates underwent a quality control process. Reads with a mean quality score <Q30 or length <36 base pairs were discarded. KRAKEN2 (*v2.1.2*) [4] was used to remove unclassified reads, and *de novo* assembly was performed with Unicycler (*v0.5.0*).

Multilocus sequence types (STs) and taxa were identified by MLST (v2.11) [5] and KRAKEN2, respectively. Drug-resistant genes were identified with two approaches. First, passed quality reads were mapped to the ResFinder database with ResFinder (v4.1.11). Second, assembled contigs were mapped to NCBI AMRFinderPlus and ResFinder databases. In this approach, ABRicate (v1.0.1) [6] was used to map assembled sequences to the NCBI AMRFinderPlus database [7] and ResFinder database [8]. All resistance genes identified were pooled, and those with more than 90% identity and coverage were selected. Additionally, plasmid replicons were identified using PlasmidFinder 2.0 (https://cge.cbs.dtu.dk/services/PlasmidFinder/).

The accession number of the isolates is PRJNA1020534 (release date: 2024-2103-01, https://www.ncbi.nlm.nih.gov/sra/PRJNA1020534); BioProject and associated SRA metadata are available at https://dataview.ncbi.nlm.nih.gov/object/PRJNA1020534?reviewer=7v8hhrtb4ago7ut598acfeq5gc.

## Results

All isolates were identified to species level *as E. coli* and *K. pneumoniae* classified by the sequence taxonomic database. Species classifications were confirmed to be correct except for two isolates SK106 and SK128 from patient numbers 24 and 29, respectively, which were reassigned from *E. coli* to *Enterobacter roggenkampii* (EER) (Supplementary Table S1). All but 7 isolates were assigned to an ST, and in total, 23 different STs were identified (Supplementary Table S1). *E. coli* isolates exhibited the greatest heterogeneity with 20 STs, whereas *K. pneumoniae* isolates were mainly ST14 and ST15. Almost all isolates from pre- and post-surgical samples shared the same ST, and isolates from 8 of the 17 patients fell in the same type. Notably, the *K. pneumoniae* isolated from patient number 30 belonged to the same genotype (ST14) as with others of this patient’s isolates but harboured different resistance genes.

Sequence analysis revealed the presence of *bla* genes in addition to other resistance genes. The most prevalent *bla*_ESBL_ genes in *E. coli* were *bla*_EC_ (69.57%), *bla*_CTX-M_ (65.22%), and *bla*_TEM_ (36.95%), whereas *bla*_SHV_ predominated in *K. pneumoniae* (41.30%) alone (Supplementary Table S2). The *bla* genes found from the isolates at pre- and post-surgery were generally of the same prevalence. Most patients, except for six individuals, had almost the same resistance gene profiles of isolates pre- and post-abdominal surgery (Supplementary Table S2).

Three classes of β-lactamases were identified: class A (*bla*_CTX-M_, *bla*_TEM_, *bla*_Z_), class C (*bla*_SHV,_ *bla*_MIR_, *bla*_DHA_), and class D (*bla*_OXA_). The most prevalent *bla*_CTX-M_, *bla*_TEM_, *bla*_SHV_, and *bla*_OXA_ variants were *bla*_CTX-M-15_, *bla*_TEM-1B_, *bla*_SHV-28_, and *bla*_OXA-1_, respectively. All *K. pneumoniae* strains harboured *bla*_SHV_ with seven different variants, namely *bla*_SHV-11_, *bla*_SHV-13_, *bla*_SHV-28_, *bla*_SHV-100_, *bla*_SHV-106_, *bla*_SHV-110_, and *bla*_SHV-187_ (Table 1).

**Table 1.** Distribution of *bla* genes among 46 strains isolated from rectal swab of patients at pre- and post-abdominal surgery

Gene	Variants	Class	Pre-surgery	Post-surgery	Total number of isolates (%)
*CTX–M*	3, 14, 15, 27, 55	An extended–spectrum β–lactamase	14	16	30 (65.22)
*TEM*	1, 1_B	A broad–spectrum β–lactamase	7	10	17 (36.95)
*SHV*	11, 13, 28, 100, 106, 110, 187	A β–lactamase	3	16	19 (41.30)
*CMY*	CMY–2	C β–lactamase	3	2	5 (10.87)
*DHA*	DHA–1	C β–lactamase	2	2	4 (8.70)
*MIR*	MIR–2, MIR–9	C β–lactamase (cephalosporin–hydrolyzing)	1	1	2 (4.35)
*CMH*	CMH–4	C β–lactamase	1	1	2 (4.35)
*EC*	5, 8, 15, 18, 19	C β–lactamase (cephalosporin–hydrolyzing)	16	16	32 (69.57)
*OXA*	OXA–1	D β–lactamase (oxacillin–hydrolyzing)	5	3	8 (17.39)

Interestingly, pAmpC β-lactamase genes, including *bla*_CMY-2_, were found in both *E. coli* and *K. pneumoniae* and *bla*_DHA-1_ in the latter. Moreover, *mcr-10.1*, mobile colistin resistance genes, were detected only in resistant *Enterobacter cloacae* (ECL^R^) (SK131 and SK132). The ESBL-producing EER carried *bla*_TEM-1B_, *bla*_CTX-M-3_, and *bla*_MIR-2_.

Various plasmid types harbouring antimicrobial resistance genes were identified such as IncFIA, IncFIB, IncFIC, IncFII, IncQ1, IncX1, IncHI2, and IncR (accession number: PRJNA1020534).

## Discussion

Different database or input sequence formats were used for sequence data analysis, leading to different drug resistance identification results. Consequently, multiple databases were used to provide more accurate data. For example, ResFinder identified the *SHV* gene when using non-assembled sequences as input, but this gene was not flagged when using assembled sequences in samples SK116, SK125, SK126, and SK127. It is possible that the process was unable to assemble the SHV sequence due to the known performance limitation of *de novo* assembly on short read data. However, the unknown ST and missing taxonomy classifications were recalled from KRAKEN2, which contained multiple taxonomical profiles of various species. Long read sequence data is therefore necessary for further approaches.

*bla*_CTX-M_, *bla*_TEM_ and *bla*_SHV_ are the most prevalent of the many ESBLs detected in various pathogens, and consequently, they have become widely disseminated across various epidemiological niches. A previous study found SHV to be distributed mostly among *K. pneumoniae* [9], and here, it was found only in this species. However, variants of the SHV type have been detected in other members of the *Enterobacterales* family and *Acinetobacter baumannii* [10, 11].

In this study, the presence of the same ST types of strains present at pre- and post-surgery was interpreted as being indicative of colonization of the patient’s gut by ESBL producers and other resistant strains before surgery. Plasmid-mediated resistance genes are readily transferable and often spread from one bacterium to another. It follows that the persistence of such strains can give rise to hazardous and difficult-to-treat post-surgical site infections. Hence, screening of patients before, and after, surgery to confirm persistent carriage of ESBL-PE strains is of practical benefit and increases clinical awareness of their potential transmission during surgery.

The multi-resistant EPEC ST131 strain has been reported worldwide due to its high risk of gastrointestinal tract infection and sometimes progression to urinary tract infection and septicaemia. It is also widely distributed as a colonist among healthy individuals and animals [3, 12, 13]. This genotype is particularly associated with several resistance genes, particularly *bla*_CTX-M_ [13]. The isolates harbouring *bla*_CMY-2_, which is the most common pAmpC β-lactamase gene reported worldwide [14], and *mcr*-*10.1* present a potentially high risk of infections during abdominal surgery in this study.

Colistin was only relatively recently introduced as the last available antibiotic for combatting multiple drug-resistant bacterial infections [15], but the presence of its resistance gene, *mcr*, in this study indicates that genetically mobilized colistin-resistant strains pose an emerging threat due to their associated high risk of morbidity and mortality. Variants of the *mcr* gene including *mcr-1* through *mcr-10* have been identified in many bacteria globally [16].

In patient number 24, an EPEC strain was isolated before surgery and an ESBL-producing EER after surgery. Both isolates were positive for *bla*_TEM-1B_ and *bla*_CTX-M-3_. These genes and *bla*_MIR-1_, a plasmid-mediated class C (group 1), confer resistance to oxyimino β-lactams. They were detected in EER, while *bla*_CMY-2_ was found in EPEC. The presence of the plasmid-mediated genes of the two species may result in their potential transfer between the strains during intestinal carriage. It is widely accepted that appropriate antibiotic use for prophylaxis is essential to reduce infections in high-risk patients. Likewise, guidelines for appropriate drug prescriptions for such individuals should be evaluated, and patients should be actively screened for carriage of ESBL producers and other resistance genes before surgery.

Extended-spectrum β-lactamase producers were not detected in 120 healthy adults as previously reported from a tertiary Thai hospital [17]. However, ESBL-producing *E. coli* and *K. pneumoniae* multidrug-resistant isolates were recently reported in approximately 30% of an elderly population living at home who had undergone abdominal surgery [18].

In conclusion, phenotypic and genotypic characteristics of a collection of isolates of ESBL-producing *E. coli and K. pneumoniae* and other plasmid-mediated resistant strains, especially mobilized colistin resistance gene *mcr*, is necessary to arrest their potential spread. This study provided detailed information on the species distribution and their resistance genes, which will aid prevention and control of post-abdominal surgical infections, and the spread of resistance genes.

## Supporting information

Kondo et al. supplementary material 1 Kondo et al. supplementary material

Kondo et al. supplementary material 2 Kondo et al. supplementary material

## Data Availability

The authors confirm that the data supporting the findings of this study are available within the article.

## Supplementary material

The supplementary material for this article can be found at http://doi.org/10.1017/S0950268824000578.
