# Supplementary material for: Genomic characterization of extended-spectrum β-lactamase-producing Enterobacterales isolated from abdominal surgical patients
Source: Epidemiol Infect. 2024 Apr 12;152:e70. doi: 10.1017/S0950268824000578 (PMC11077598; doi:10.1017/S0950268824000578)
Supplement: Kondo et al. supplementary material 1 — Kondo et al. supplementary material [file S0950268824000578sup001.docx]

'Epidemiology and Infection'

Genomic characterization of extended-spectrum β-lactamase-producing *Enterobacterales* isolated from abdominal surgical patients

Kondo, S, Phornsiricharoenphant, W, Na-rachasima, L, Phokhaphan, P, Ruangchai, W, Palittapongarnpim P, Apisarnthanarak, A

'Supplementary Material'

**Supplementary Table S1** Sequence types (ST) of ESBL-producing *Enterobacterales* and resistant strains isolated from patients who underwent abdominal surgery

| **Patient** |  | **Strain code** | **Isolate*** | **Pre (0)/ post (1)** |  | **ST** |
| --- | --- | --- | --- | --- | --- | --- |
| 3 |  | SK3 | EPE | 0 |  | 46/398 |
|  |  | SK76 | EPE | 1 |  | 1324/- |
|  |  | SK77 | EPE | 1 |  | 1324/- |
| 4 |  | SK4 | EPE | 0 |  | 7506/730 |
|  |  | SK6 | EPE | 1 |  | 131/506 |
| 5 |  | SK8 | EP^R^ | 0 |  | 537/470 |
|  |  | SK11 | EPE | 0 |  | 654/- |
|  |  | SK90 | EP^R^ | 1 |  | 537/470 |
| 9 |  | SK20 | EPE | 0 |  | 38/535 |
|  |  | SK23 | EPE | 1 |  | 38/535 |
| 13 |  | SK80 | EPE | 0 |  | 131/43 |
|  |  | SK81 | EPE | 1 |  | 131/43 |
| 14 |  | SK82 | EPE | 0 |  | Unknown |
|  |  | SK83 | EPE | 1 |  | 457/- |
| 16 |  | SK85 | EKP | 1 |  | Unknown |
|  |  | SK87 | EP^R^ | 0 |  | Unknown |
| 17 |  | SK88 | EPE | 0 |  | 131/43 |
|  |  | SK89 | EPE | 1 |  | 131/43 |
| 18 |  | SK91 | EPE | 0 |  | 405/477 |
|  |  | SK92 | EPE | 1 |  | 405/477 |
| 20 |  | SK96 | EPE | 0 |  | 2/3171 |
|  |  | SK100 | EPE | 1 |  | 405/44 |
|  |  | SK95 | EKP | 0 |  | 15 |
|  |  | SK97 | EKP | 0 |  | 15 |
|  |  | SK98 | KP^R^ | 1 |  | 15 |
|  |  | SK99 | KP^R^ | 1 |  | 15 |
| 22 |  | SK101 | EPE | 0 |  | 871/- |
|  |  | SK102 | EPE | 1 |  | 871/- |
| 23 |  | SK103 | EPE | 0 |  | 4014/88 |
|  |  | SK104 | EKP | 1 |  | 37 |
| 24 |  | SK105 | EPE | 0 |  | 648/- |
|  |  | SK106 | EER | 1 |  | Unknown |
| 27 |  | SK109 | EPE | 0 |  | 1485/- |
|  |  | SK110 | EPE | 0 |  | 1193/53 |
|  |  | SK111 | EPE | 1 |  | 1193/53 |
|  |  | SK112 | EPE | 1 |  | 1193/53 |
| 29 |  | SK114 | EPE | 1 |  | 773/- |
|  |  | SK128 | ER^R^ | 0 |  | Unknown |
| 30 |  | SK116 | KP^R^ | 1 |  | 14 |
|  |  | SK125 | KP^R^ | 1 |  | 14 |
|  |  | SK126 | EKP | 1 |  | 14 |
|  |  | SK127 | KP^R^ | 1 |  | 14 |
| 35 |  | SK129 | EP^R^ | 1 |  | 648/- |
|  |  | SK130 | EPE | 1 |  | 1193/53 |
|  |  | SK131 | ECL^R^ | 0 |  | Unknown |
|  |  | SK132 | ECL^R^ | 1 |  | Unknown |

* EPE = ESBL-producing *E. coli*, EP^R^ = resistant *E. coli*, EKP = ESBL-producing *K. pneumoniae*, KP^R^ = resistant *K. pneumoniae*, ECL^R^ = resistant *Enterobacter cloacae*, EER = ESBL-producing *Enterobacter roggenkampii*.
