# Supplementary material for: Genomic characterization of extended-spectrum β-lactamase-producing Enterobacterales isolated from abdominal surgical patients
Source: Epidemiol Infect. 2024 Apr 12;152:e70. doi: 10.1017/S0950268824000578 (PMC11077598; doi:10.1017/S0950268824000578)
Supplement: Kondo et al. supplementary material 2 — Kondo et al. supplementary material [file S0950268824000578sup002.docx]

'Epidemiology and Infection'

Genomic characterization of extended-spectrum β-lactamase-producing *Enterobacterales* isolated from abdominal surgical patients

Kondo, S, Phornsiricharoenphant, W, Na-rachasima, L, Phokhaphan, P, Ruangchai, W, Palittapongarnpim P, Apisarnthanarak, A

'Supplementary Material'

**Supplementary Table S2** Genotypic characteristics among strains isolated from rectal swab of the patients at pre- and post- abdominal surgery

| **Patient No.** | **Ward** | **Strain code** | **Isolate** | **Pre (0)/ post (1) surgery** | **ST** |  | ***bla* genes** |  | **Other resistance genes** |
| --- | --- | --- | --- | --- | --- | --- | --- | --- | --- |
| 3 | 1 | SK3 | EPE | 0 | 46/398 |  | TEM-1B,  CTX-M14,  EC-15 |  | *aadA2, tet(A), aph(6)-Id, aph(3'')-Id, lnu(F),qnrS1, floR, sul3, dfrA14* |
|  |  | SK76 | EPE | 1 | 1324/- |  | CTX-M14,  TEM-1B,  EC-18 |  | *aadA1, aac(3)-Iid, aadA2, lnu(F), floR, tet(A), sul3* |
|  |  | SK77 | EPE | 1 | 1324/- |  | CTX-M14,  TEM-1B,  EC-18 |  | *aac(3)-Iid, aadA2, lnu(F), floR, tet(A)* |
| 4 | 1* | SK4 | EPE | 0 | 7506/730 |  | CMY-2,  EC-18 |  | *tet(A), aph(6)-Id, aph(3'')-Ib, aadA2, sul2, oqxA, oqxB, floR, lnu(F), erm(42), bleO* |
|  |  | SK6 | EPE | 1 | 131/506 |  | TEM-1B,  CTX-M27,  EC-5 |  | *mph(A), sul1, sul2, dfrA17* |
| 5 | 1 | SK8 | EP^R^ | 0 | 537/470 |  | CMY-2,  EC-5 |  |  |
|  |  | SK11 | EPE | 0 | 654/- |  | CTX-M14,  EC-18 |  | *mph(A), qnrS1,aadA2, cmlA1, aadA1, aac(3)-Iid, sul3, dfrA12* |
|  |  | SK90 | EP^R^ | 1 | 537/470 |  | CMY-2,  EC-5 |  | None |
| 9 | 2* | SK20 | EPE | 0 | 38/535 |  | CTX-M15,  EC-8 |  | *qnrS1, tet(A)* |
|  |  | SK23 | EPE | 1 | 38/535 |  | CTX-M15,  EC-8 |  | *qnrS1, tet(A)* |
| 13 | 1 | SK80 | EPE | 0 | 131/43 |  | CTX-M15,  EC-5,  OXA-1 |  | *tet(A), aac(3)-Iie, aac(6')-Ib-D181Y* |
|  |  | SK81 | EPE | 1 | 131/43 |  | CTX-M15,  EC-5,  OXA-1 |  | *tet(A), aac(3)-Iie, aac(6')-Ib-D181Y* |
| 14 | 1 | SK82 | EPE | 0 | Unknown |  | CTX-M55,  EC |  | *tet(A), aph(6)-Id, aph(3'')-Ib, qnrS1, aac(3)-Iid, mph(A), sul3, arr-2, aph(3')-Ia, sul2, dfrA14* |
|  |  | SK83 | EPE | 1 | 457/- |  | CTX-M55,  EC-8 |  | *qnrS1, tet(A), cmlA1, aadA1, floR, sul2, sul3, aph(3')-Ia, lnu(F), aac(3)-Iid, aadA2, dfrA12* |
| 16 | 2* | SK85 | EKP | 1 | Unknown |  | SHV-110 |  | *oqxA6, oqxB31, fosA_gen* |
|  |  | SK87 | EP^R^ | 0 | Unknown |  | TEM-1,  CTX-M15,  OXA-1,  EC-18 |  | *erm(B), mph(A), aac(3)-Iie, aac(6')-Ib-D181Y* |
| 17 | 1* | SK88 | EPE | 0 | 131/43 |  | CTX-M14,  TEM-1B,  EC-5 |  | *aac(3)-Iid, qnrS1, aadA5, sul1, sul2, aph(3'')-Ib, aph(6)-Id, tet(A), erm(B), mph(A), dfrA17* |
|  |  | SK89 | EPE | 1 | 131/43 |  | CTX-M14,  TEM-1B,  EC-5 |  | *aac(3)-Iid, qnrS1, aadA5, sul1, sul2, aph(3'')-Ib, aph(6)-Id, tet(A), erm(B), mph(A), dfrA17* |
| 18 | 1 | SK91 | EPE | 0 | 405/477 |  | CTX-M15,  EC-8,  OXA-1 |  | *dfrA17, tet(B), sul1, aadA5, mph(A), aac(6')-Ib-D181Y* |
|  |  | SK92 | EPE | 1 | 405/477 |  | CTX-M15,  EC-8,  OXA-1 |  | *tet(B), sul1, aadA5, mph(A), aac(6')-Ib-D181Y, dfrA17* |
| 20 | 1 | SK96 | EPE | 0 | 2/3171 |  | CTX-M15,  EC |  | *floR* |
|  |  | SK100 | EPE | 1 | 405/44 |  | CTX-M55,  EC-8 |  | *aac(3)-Iid, catA2,aadA5, sul1, sul2, mph(A), qnrS1, aph(3'')-Ib, aph(6)-Id, tet(A), erm(B), dfrA17* |
|  |  | SK95 | EKP | 0 | 15 |  | SHV-106,  SHV-28^**^,  DHA-1,  OXA-1 |  | *oqxA6, oqxB20, fosA6, sul1, qnrB4, catB3, aac(6')-Ib-cr5, tet(A), aac(3)-Iid, catA2* |
|  |  | SK97 | EKP | 0 | 15 |  | SHV-106,  DHA-1,  OXA-1 |  | *oqxA6, oqxB20, fosA6, sul1, qnrB4, catB3, aac(6')-Ib-cr5, tet(A), aac(3)-Iid, catA2* |
|  |  | SK98 | KP^R^ | 1 | 15 |  | SHV -106,  SHV-28^**^,  DHA-1,  OXA-1 |  | *oqxA6, oqxB20, fosA6, sul1, qnrB4, catB3, aac(6')-Ib-cr5, tet(A), aac(3)-Iid, catA2* |
|  |  | SK99 | KP^R^ | 1 | 15 |  | SHV-106 |  | *oqxA6, oqxB20, fosA6* |
| 22 | 1 | SK101 | EPE | 0 | 871/- |  | TEM-1B,  CTX-M-55,  EC-15 |  | *qnrS1, sul2, sul3, aac(3)-Iid, aadA25, aph(3')-Ia, aph(3'')-Ib, mef(B), floR, dfrA12* |
|  |  | SK102 | EPE | 1 | 871/- |  | TEM-1B,  CTX-M55,  EC-15 |  | *qnrS1, sul2, sul3, aac(3)-Iid, aadA25, aph(3')-Ia, aph(3'')-Ib, mef(B), floR, dfrA12* |
| 23 | 3* | SK103 | EPE | 0 | 4014/88 |  | TEM-1B,  CTX-M55,  EC-18 |  | *tet(X), floR* |
|  |  | SK104 | EKP | 1 | 37 |  | SHV-187 |  | *oqxA6, oqxB22, fosA6* |
| 24 | 3 | SK105 | EPE | 0 | 648/- |  | TEM-1B,  CTX-M-3,  CMY-2^**^,  EC-19 |  | *tet(A), dfrA17* |
|  |  | SK106 | EER | 1 | Unknown |  | TEM-1B,  CTX-M-3,  MIR-9,  MIR-2^**^ |  | *oqxB20, oqxA10, fosA* |
| 27 | 3* | SK109 | EPE | 0 | 1485/- |  | TEM-1B,  CTX-M55,  EC-19 |  | *qnrS13, sul2, aph(3'')-Ib, aph(6)-Id* |
|  |  | SK110 | EPE | 0 | 1193/53 |  | CTX-M27,  EC-5 |  | *tet(A), mph(A), sul1, sul2, aadA5, aph(6)-Id, aph(3'')-Ib, dfrA17* |
|  |  | SK111 | EPE | 1 | 1193/53 |  | CTX-M27,  EC-5 |  | *tet(A), mph(A), sul1, sul2, aadA5, aph(6)-Id, aph(3'')-Ib, dfrA17* |
|  |  | SK112 | EPE | 1 | 1193/53 |  | CTX-M27,  EC-5 |  | *tet(A), mph(A), sul1, sul2, aadA5, aph(6)-Id, aph(3'')-Ib, dfrA17* |
| 29 | 1 | SK114 | EPE | 1 | 773/- |  | TEM-1B,  CTX-M14,  EC-15 |  | *tet(A), tet(B), tet(X), aph(3'')-Ib, sul2, lnu(F), aadA2, aac(3)-Iid, floR, catA1, dfrA17* |
|  |  | SK128 | ER^R^ | 0 | Unknown |  | MIR-9 |  | *fosA, oqxB9, oqxA10* |
| 30 | 1* | SK116 | KP^R^ | 1 | 14 |  | SHV -11,  SHV -28 |  | *oqxA6, oqxB20, erm (c), fosA6* |
|  |  | SK125 | KP^R^ | 1 | 14 |  | SHV-11,  SHV-28^**^,  *bla*Z^**^ |  | *oqxA6, oqxB20, erm (c), fosA6* |
|  |  | SK126 | EKP | 1 | 14 |  | SHV-11^**^,  SHV-28^**^,  SHV-13^**^ |  | *oqxA6, oqxB20, erm (c), fosA6* |
|  |  | SK127 | KP^R^ | 1 | 14 |  | SHV-100^**^,  SHV-106 |  | *oqxA6, oqxB20, fosA6* |
| 35 | 4 | SK129 | EP^R^ | 1 | 648/- |  | CTX-M27,  CMY-2,  EC-19, |  | *sul1, sul2, aph(3'')-Ib, aph(6)-Id, aadA5, tet(A), tet(B), floR* |
|  |  | SK130 | EPE | 1 | 1193/53 |  | TEM-1 |  | *mph(A), sul1, sul2, aadA5, aac(3)-Iid, dfrA17, aph(3'')-Ib, aph(6)-Id, tet(A)* |
|  |  | SK131 | ECL^R^ | 0 | Unknown |  | CMH-4 |  | *oqxA10, oqxB9, mcr-10.1* |
|  |  | SK132 | ECL^R^ | 1 | Unknown |  | CMH-4 |  | *oqxA10, oqxB9, mcr-10.1* |

*1 = General Surgery Ward; 1* = Special Surgery Ward 1; 2 = Special Surgery Ward 2; 3, 3*, 4 = Special Hospital Ward

** Genes that only found by Resfinder based on non-assembled sequences.
